# Tamoxifen treatment reverses the adverse effects of chemotherapy-induced ovarian failure on serum lipids

**DOI:** 10.1038/sj.bjc.6601979

**Published:** 2004-07-13

**Authors:** L Vehmanen, T Saarto, C Blomqvist, M-R Taskinen, I Elomaa

**Affiliations:** 1 Department of Oncology, Helsinki University Central Hospital, PO BOX 180, HUCH, FIN-00290, Helsinki, Finland; 2 Department of Medicine, Helsinki University Central Hospital, FIN-00290, Helsinki, Finland

**Keywords:** breast cancer, chemotherapy, tamoxifen, serum lipids

## Abstract

In all, 146 premenopausal women with early stage breast cancer were treated with adjuvant chemotherapy. In addition, 5-year tamoxifen treatment was started after chemotherapy to those 112 patients with hormone-receptor-positive tumours while those with hormone-receptor-negative tumours received no further therapy. The serum lipid levels were followed in both groups. The levels of serum total and low-density lipoprotein (LDL) cholesterol increased significantly after chemotherapy only in patients who developed ovarian dysfunction. Total cholesterol increased +9.5% and LDL cholesterol +16.6% in patients who developed amenorrhoea (*P*<0.00001 and 0.00001, respectively). The cholesterol levels did not change in patients who preserved regular menstruation after chemotherapy. After 6 months of tamoxifen therapy, the total cholesterol decreased −9.7% and the LDL cholesterol −16.7% from levels after the chemotherapy, while the cholesterol concentrations remained at increased levels in the control group (*P*=0.001 and *P*<0.0001, respectively). The high-density lipoprotein cholesterol levels did not change significantly in either tamoxifen or control group. The effects of tamoxifen treatment on serum lipids after chemotherapy have not been studied before. Our current study suggests that adjuvant tamoxifen therapy reverses the adverse effects of chemotherapy-induced ovarian failure on total and LDL cholesterol and even lowers their serum levels below the baseline.

Adjuvant chemotherapy significantly improves survival of premenopausal and perimenopausal breast cancer patients (Early Breast Cancer Trialists‘ Collaborative Group, 1998a). In a majority of these patients, however, adjuvant chemotherapy causes ovarian failure. The incidence of adjuvant chemotherapy induced amenorrhoea varies from 26 to 89% depending on the drug combination used (Del Mastro *et al*, 1997). Most data are available on regimens based on cyclophosphamide, methotrexate and fluorouracil (CMF) and the average rate of CMF-induced ovarian failure is 68% (Bines *et al*, 1996). Women most prone to develop ovarian failure are those in their 40s, while women under 40 years of age have better preservation of menstruation after combination chemotherapy (Koyama *et al*, 1977; Bonadonna *et al*, 1985; Ludwig Breast Cancer Study Group, 1985; Padmanabhan *et al*, 1987; Richards *et al*, 1990; Bianco *et al*, 1991; Bines *et al*, 1996).

The effects of chemotherapy on serum lipids in premenopausal women with breast cancer have been somewhat conflicting in the few studies available on subject. The levels of low-density lipoprotein (LDL) and high-density lipoprotein (HDL) cholesterol decreased and total cholesterol increased only slightly after chemotherapy in one study (Rzymowska, 1999), while in another study HDL cholesterol levels increased and total cholesterol levels decreased (Subramaniam *et al*, 1991). We have previously shown that the changes in serum lipids after adjuvant chemotherapy correlate to the changes in menstruation: total cholesterol, LDL and HDL cholesterol levels increased significantly only in patients with chemotherapy-induced ovarian dysfunction, while patients who preserved menstruation had no changes in these serum lipid levels (Saarto *et al*, 1996a). The effects of chemotherapy on serum triglyceride levels have varied across the studies (Subramaniam *et al*, 1991; Saarto *et al*, 1996a; Rzymowska, 1999).

Both natural and surgical menopause cause changes in serum lipids that are explained by the deficiency of oestrogens: serum total and LDL cholesterol and triglyceride levels increase and HDL cholesterol levels decrease (Matthews *et al*, 1989; Newnhamn 1993; Stevenson *et al*, 1993; Schaefer *et al*, 1994; Fukami *et al*, 1995). These adverse effects of menopause on serum lipids are reversed by hormone replacement therapy (Rijpkema *et al*, 1990; Nabulsi *et al*, 1993; Newnhamn, 1993; Writing Group for the PEPI Trial, 1995; Pripp *et al*, 1999). A few observational trials suggested a protective effect against coronary heart disease (CHD) among users of oestrogen or combined oestrogen and progestin (Stampfer and Colditz, 1991; Grady *et al*, 1992). However, two more recent large randomised trials have failed to show any cardioprotection among hormone replacement therapy users (Hulley *et al*, 1998; Manson *et al* 2003). The effects of tamoxifen on serum lipids have been extensively studied. Uniformly, the levels of total cholesterol and LDL cholesterol decrease significantly during tamoxifen treatment, but the effects on HDL cholesterol and triglycerides have varied (Love *et al*, 1990; Grey *et al*, 1995; Saarto *et al*, 1996b; Decensi *et al*, 1998). Two retrospective, randomised trials suggested that tamoxifen might have a cardioprotective effect in postmenopausal women (McDonald and Stewart, 1991; Rutqvist and Mattsson, 1993). However, a large placebo-controlled randomised study failed to show any effects of tamoxifen on cardiovascular risk (Fisher *et al*, 1998).

Adjuvant tamoxifen treatment given after chemotherapy extends disease-free and overall survival for oestrogen receptor-positive breast cancer (Early Breast Cancer Trialists‘ Collaborative Group, 1992; 1998b). So far no studies are available on the effects of chemotherapy followed by tamoxifen on serum lipid levels. The aim of this study was to investigate whether tamoxifen treatment after chemotherapy could reverse the adverse effects of chemotherapy on serum lipid levels in premenopausal women with breast cancer.

## MATERIALS AND METHODS

### Patients

The study population consists of 159 premenopausal newly diagnosed breast cancer patients with operable T1-3 N0-2 M0 breast cancer, treated between January 1998 and May 2001 at Helsinki University Hospital, Department of Oncology. Exclusion criteria were the following: (1) Karnofsky performance index <70, (2) hysterectomy or bilateral ovariectomy, (3) pregnancy or lactation, (4) age >55 years, (5) untreated hypothyreosis or hyperthyreosis and (6) other malignancies. Premenopausal status was defined as ongoing menstruation during the last 6 months. Four patients used a levonorgestrel-releasing intrauterine system (LNG IUS) as a contraceptive device at entry and therefore had sparse menstruation; baseline levels of follicle-stimulating hormone (FSH) were within premenopausal range (4.2–18.9 IU l^−1^) also in these patients.

Of the 159 patients at entry, 13 patients were excluded. Four patients developed distant metastases before 1 year of follow-up, four patients discontinued follow-up, three patients had medication affecting lipid metabolism, one patient had missing baseline lipid values and one patient was diagnosed with untreated hypothyreosis. Overall, 146 patients were eligible for analyses.

All patients underwent surgery with axillary evacuation and total mastectomy or breast-conserving resection. Postoperative radiotherapy was given to those treated with breast-conserving surgery and to those diagnosed with axillary lymph node metastases. All patients were treated with adjuvant chemotherapy. The standard adjuvant chemotherapy regimen at Helsinki University Hospital was changed during the study period. Consequently, 73 patients received six cycles of cyclophosphamide (600 mg m^−2^), methotrexate (40 mg m^−2^) and 5-fluorouracil (600 mg m^−2^) intravenously on day 1 with 3 weeks' intervals and one cycle of cyclophosphamide during the radiotherapy (CMF). In total, 72 patients received six to nine cycles of cyclophosphamide (600 mg m^−2^), epirubicin (60 mg m^−2^) and 5-fluorouracil (600 mg m^−2^) intravenously similarly with 3 weeks' intervals (CEF) while one patient received four cycles of cyclophosphamide (600 mg m^−2^) and adriamycin (60 mg m^−2^). All patients were treated with a prophylactic antiemetic regimen typically consisting of a serotonin (HT3) receptor antagonist (tropisetron or granisetron), metoclopramide and dexamethasone. After the chemotherapy, adjuvant 5-year tamoxifen was recommended to hormone-receptor-positive patients.

In addition, the first 48 patients were randomly allocated to receive intermittent intravenous clodronate treatment or no further therapy. A measure of 1500 mg of clodronate was given in saline over 3 h before each chemotherapy infusion for seven consecutive cycles. Randomisation was later interrupted due to conflicting results of adjuvant clodronate trials (Diel *et al*, 1998; Saarto *et al*, 2001; Powles *et al*, 2002).

### Methods

Informed consent was obtained from all participants. The study was approved by the Local Ethical Committee, at the Department of Oncology, at the Helsinki University Hospital. Staging investigations for breast cancer included clinical investigation, liver ultrasound and bone scintigraphy. Basic laboratory tests before randomisation included a complete blood count and sedimentation rate, liver enzymes (transaminase, alkaline phosphatase, 5-nucleotidase), serum creatinine, albumin, calcium and kalium. Patients were interviewed regarding menopausal status, medications and other diseases before randomisation and at 6 and 12 months. Permanent amenorrhoea was defined as absent menstruation for at least 6 months. At 12 months, the patients were divided into three groups with respect to menstrual function after chemotherapy (regular menstruation, irregular menstruation and amenorrhoea). The following measurements were analysed from the fasting blood samples before the initiation of therapy and at 3, 6, 9 and 12 months: serum concentrations of total cholesterol, LDL cholesterol, HDL cholesterol, triglycerides, serum FSH, luteinizing hormone (LH) and oestradiol.

The serum cholesterol level was determined with an enzymatic colorimetric CHOD-PAP method and the triglyceride level with an enzymatic colorimetric GPO-PAP method (Roche Diagnostics). The concentration of HDL cholesterol was measured by an enzymatic HDL-C plus second-generation method (Roche Diagnostics). The equipment used to measure serum cholesterol, HDL-cholesterol and triglyceride levels was Hitachi 917 or Modular analysator (Hitachi Ltd, Tokyo, Japan). LDL cholesterol was calculated according to Friedewald equation (LDL cholesterol=cholesterol − HDL cholesterol − Trigly/2.2) (Friedewald *et al*, 1972).

### Statistical methods

The Wilcoxon matched pair test was used to compare lipid and hormonal changes from baseline to 6 months within each menstrual group (regular menses, irregular menses and amenorrhoea). The Wilcoxon matched pair test was also used to test lipid changes from 6 to 9 months and from 6 to 12 months within the tamoxifen and control groups. The differences between the menstrual groups were tested by a repeated measures ANOVA model using the programs Statistical Package for the Social Science (SPSS) for Macintosh. Similarly, the effects of tamoxifen and clodronate treatment on changes in serum lipids were tested by repeated measures ANOVA. The correlations between the changes in serum lipids and weight were assessed by Spearman's rank-order correlation coefficient. Other comparisons were made using the Mann–Whitney test. Owing to multiple comparisons, the significance level was set at 0.01.

## RESULTS

### Responses of serum lipids to chemotherapy-induced ovarian dysfunction (from baseline to 6 months)

Chemotherapy caused amenorrhoea in the majority of the patients. During 1 year follow-up, 78 patients of 146 (53%) had developed permanent amenorrhoea (absence of menstruation for at least 6 months) and 47 (32%) had irregular menstruation, while only 21 patients (14%) had regular menstruation. The mean age of the patients at the start of the chemotherapy was 47 years for those who developed amenorrhoea, 41 years for those with irregular menstruation and 36 years for those who preserved regular menstruation, respectively.

The gonadotropin FSH and LH changes during the chemotherapy period correlated with the changes observed in menstrual cycle. In patients who developed amenorrhoea, the median value of FSH rose from 5.45 to 71.10 IU l^−1^ (*P*<0.00001). The median FSH value rose from 5.30 to 47.90 IU l^−1^ (*P*<0.00001) in patients with irregular menstruation and from 5.10 to 19.60 IU l^−1^ (*P*=0.0001) in those who preserved regular menstruation. Similarly, the median LH values rose significantly during the chemotherapy in each menstrual group (Table 1

**Table 1:** Hormonal and lipid levels after the chemotherapy according to menstrual status at 12 months

	**Regular menses**			**Irregular menses**			**Amenorrhoea**		
	**Baseline**	**3 months**	**6 months**	**Baseline**	**3 months**	**6 months**	**Baseline**	**3 months**	**6 months**
FSH (IU l^−1^)*	5.10 (1.40–11.40)	8.30 (2.10–53.10)^a^	19.60 (4.50–83.40)^b^	5.30 (1.40–39.30)	23.50 (2.40–64.50)^c^	47.90 (2.00–96.10)^c^, ^c^	5.45 (1.60–96.60)	65.60 (5.90–146.00)^c^	71.10 (31.00–146.00)^c^, ^d^
LH (IU l^−1^)*	4.80 (0.60–15.0)	7.00 (3.40–36.70)	20.50 (4.10–58.30)^b^	4.20 (1.00–37.60)	16.10 (2.40–43.40)^c^	28.10 (0.70–56.40)^c^, ^e^	4.80 (0.70–50.00)	36.25 (5.50–70.50)^c^	33.80 (15.20–74.40)^c^, ^d^
Oestradiol (nmol l^−1^)*	0.24 (0.04–0.91)	0.18 (0.03–0.65)	0.20 (0.02–0.84)	0.20 (0.03–1.29)	0.17 (0.02–0.94)	0.08 (0.02–1.26)^e^	0.28 (0.02–1.20)	0.02 (0.02–0.75)^c^	0.02 (0.02–0.29)^d^, ^e^
Cholesterol (mmol l^−1^)**	5.22 (0.80)	5.25 (0.79)	5.31 (0.84)	5.00 (0.82)	5.31 (0.89)^a^	5.33 (0.98)^a^	5.44 (0.83)	5.80 (0.96)^b^	5.91 (0.98)^c^
LDL (mmol l^−1^)**	3.16 (0.69)	3.04 (0.76)	3.22 (0.78)	2.83 (0.79)	3.02 (0.83)^a^	3.08 (0.85)	3.07 (0.72)	3.31 (0.93)^b^	3.53 (0.92)^c^
HDL (mmol l^−1^)**	1.64 (0.29)	1.68 (0.34)	1.61 (0.33)	1.77 (0.40)	1.80 (0.48)	1.75 (0.44)	1.90 (0.43)	1.91 (0.49)	1.85 (0.40)
Triglyceride (mmol l^−1^)**	0.94 (0.50)	1.15 (0.73)	1.04 (0.51)	0.89 (0.32)	1.09 (0.59)^a^	1.08 (0.60)	1.03 (0.53)	1.26 (0.72)^b^	1.16 (0.57)^a^
LDL/HDL**	2.02 (0.67)	1.93 (0.75)	2.12 (0.78)	1.70 (0.65)	1.83 (0.77)	1.89 (0.74)	1.72 (0.62)	1.88 (0.80)^a^	2.01 (0.70)^c^
Cholesterol/HDL**	3.29 (0.83)	3.28 (0.96)	3.43 (0.90)	2.94 (0.73)	3.13 (0.91)	3.21 (0.93)^a^	2.98 (0.74)	3.23 (1.02)^a^	3.32 (0.82)^c^

* Median (range),

** Mean (s.d.). Significance of the changes from baseline to 3 and 6 months within the groups.

a *P*<0.01.

b *P*<0.001.

c *P*<0.00001. None of the differences between regular and irregular menses groups in changes from baseline to 6 months were statistically significant. Differences between regular menses and amenorrhoea groups in changes from baseline to 6 months:

d *P*<0.00001. Differences between irregular menses and amenorrhoea groups in changes from baseline to 6 months:

e *P*<0.00001.

).

Changes in total and LDL cholesterol during the chemotherapy correlated significantly with menstrual function. Only those patients who developed either amenorrhoea or irregular menstruation had marked elevations in serum total and LDL cholesterol, while no significant changes occurred in those who preserved regular menstruation.

In patients who developed amenorrhoea, the total cholesterol increased by +9.5% and the LDL cholesterol by +16.6% (*P*<0.00001 and 0.00001, respectively). The LDL/HDL ratio increased by 21.7% (*P*<0.00001) and the total cholesterol/HDL ratio by +13.3% (*P*<0.00001).

The total cholesterol increased by +7.3% and LDL cholesterol by +11.8% in patients with irregular menstruation (*P*=0.003 and 0.017, respectively). The LDL/HDL ratio increased by +14.7% (*P*=0.02) and the cholesterol/HDL ratio +9.4% (*P*=0.005).

In patients who preserved regular menstruation, the total cholesterol increased only +2.4% and the LDL cholesterol +3.0% (*P*=0.52 and 0.57, respectively). Accordingly, LDL/HDL cholesterol and cholesterol/HDL cholesterol ratios remained unchanged (Table 1).

The differences in the changes of serum total and LDL cholesterol were insignificant between patients with amenorrhoea and irregular menstruation (*P*=0.61 and 0.22, respectively), but the differences in the changes of serum total and LDL cholesterol between patients with regular menses and irregular or absent menstruation (amenorrhoea) were more marked (*P*=0.04 and 0.008). Similarly, the differences in the changes of LDL/HDL ratios and total cholesterol/HDL ratios were insignificant between patients with amenorrhoea and irregular menstruation (*P*=0.50 and 0.84), but the differences in the changes of LDL/HDL ratios were significant (*P*=0.006) and of total cholesterol/HDL ratios nearly significant (*P*=0.02) between patients with regular menses and irregular or absent menstruation (amenorrhoea). Serum triglyceride levels increased and HDL cholesterol levels slightly decreased regardless of menstrual function and the differences between the groups were statistically insignificant (Table 1).

The mean weight gain during the chemotherapy was 2.5 kg for those who preserved regular menstruation, 2.0 kg for those with irregular menstruation and 1.2 kg for those with amenorrhoea. No correlation was found between the changes in weight and serum lipids (total cholesterol, LDL cholesterol, HDL cholesterol and triglycerides).

### Effect of adjuvant chemotherapy followed by tamoxifen on serum lipids (from 6 to 12 months)

After the chemotherapy period at 6 months, adjuvant 5-year tamoxifen was started to those 112 patients with hormone-receptor-positive tumours (tamoxifen group). In all, 34 patients with hormone-receptor-negative tumours received no further medication (control group). The patient characteristics (age, weight, body mass index, serum FSH, LH and oestradiol) were well balanced in the tamoxifen and control groups as well as the baseline values of the serum lipids (Table 2

**Table 2:** Lipid levels in the tamoxifen and control groups

	**Tamoxifen (*n*=112)**				**Control (*n*=34)**			
	**Baseline**	**6 months**	**9 months**	**12 months**	**Baseline**	**6 months**	**9 months**	**12 months**
Cholesterol (mmol l^−1^)*	5.29 (0.78)	5.60 (0.90)	5.02 (0.91)^a^	5.02 (0.85)^a^	5.18 (1.00)	5.75 (1.28)	5.69 (1.04)^b^	5.58 (1.03)^b^
LDL (mmol l^−1^)*	3.02 (0.71)	3.30 (0.80)	2.73 (0.83)^a^	2.72 (0.78)^a^	2.97 (0.88)	3.46 (1.17)	3.41 (0.85)^c^	3.32 (0.87)^c^
HDL (mmol l^−1^)*	1.83 (0.43)	1.78 (0.42)	1.77 (0.49)	1.77 (0.44)	1.79 (0.36)	1.80 (0.39)	1.81 (0.44)	1.79 (0.43)
Triglyceride (mmol l^−1^)*	0.98 (0.47)	1.13 (0.58)	1.15 (0.69)	1.16 (0.63)	0.92 (0.46)	1.08 (0.54)	1.02 (0.50)	1.02 (0.50)
LDL/HDL*	1.76 (0.62)	1.98 (0.70)	1.67 (0.75)^a^	1.66 (0.70)^a^	1.74 (0.71)	2.02 (0.82)	1.98 (0.64)^b^	1.97 (0.68)^b^
Cholesterol/HDL*	3.02 (0.73)	3.30 (0.85)	3.01 (0.91)^a^	3.00 (0.85)^a^	2.99 (0.84)	3.31 (0.94)	3.26 (0.77)	3.26 (0.85)

* Mean (s.d.). Significance of the changes from 6 to 9 months and from 6 to 12 months within the tamoxifen and control groups:

a *P*<0.00001. Differences between the tamoxifen and control groups in changes from 6 to 9 months and from 6 to 12 months:

b *P*<0.01.

c *P*<0.0001.

). All differences in these pretreatment characteristics between the treatment groups were statistically nonsignificant. Overall, 67% of the women who continued to menstruate regularly after the chemotherapy, 77% of the women who developed irregular menstruation and 79% of the women who became amenorrhoeic went on to tamoxifen. The differences in the relative proportions of the women starting tamoxifen were insignificant between the three menstrual groups (*P*=0.29). In the control group, the changes seen in the serum FSH, LH, oestradiol and lipid values from 6 to 12 months did not differ between the three menstrual groups (regular menstruation, irregular menstruation and amenorrhoea).

The total and LDL cholesterol and triglyceride levels increased during the chemotherapy in all patients. After 3 and 6 months of tamoxifen treatment, the total cholesterol decreased by −9.6 and −9.7% from the levels after chemotherapy, while total cholesterol remained unchanged in the control group (*P*=0.001 and 0.001, respectively). The LDL cholesterol decreased by −16.0 and −16.7% after 3 and 6 months of tamoxifen treatment while in the control group the LDL cholesterol did not change (*P*<0.0001 and 0.0001, respectively). The changes in HDL cholesterol levels after 3 and 6 months of tamoxifen therapy were insignificant both in the tamoxifen group and in the control group (*P*=0.78 and 0.94, respectively). The serum triglyceride remained at an increased level in both groups (Table 2).

After 3 and 6 months of tamoxifen treatment, the LDL/HDL ratio decreased −14.7 and −15.0% from the levels after the chemotherapy, while the LDL/HDL ratio remained at an increased level in the control group (*P*=0.006 and 0.003, respectively). A similar trend was observed for the total cholesterol/HDL ratio which decreased by −8.4 and −8.6% after 3 and 6 months of tamoxifen treatment, while the changes were marginal in the control patients (*P*=0.02 and 0.02, respectively) (Table 2).

**Figure 1:**
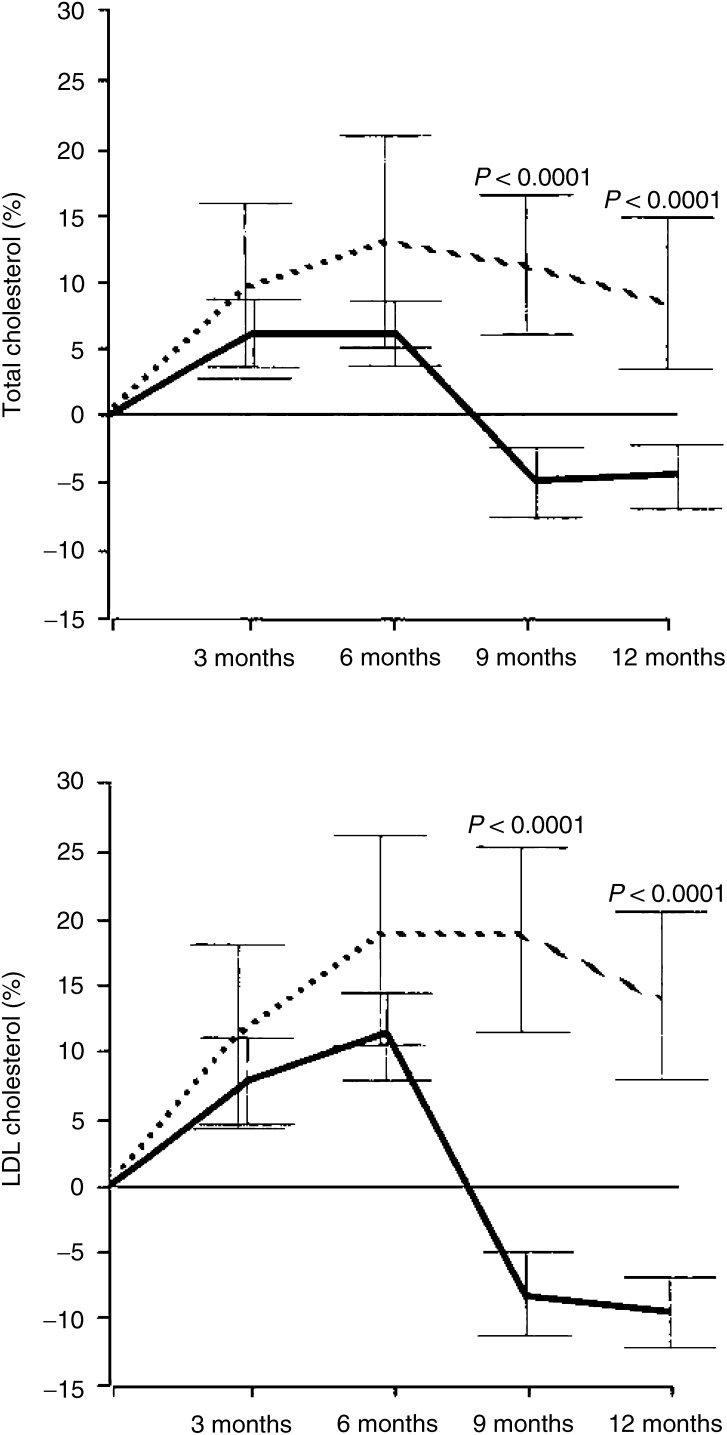
Percentual changes from baseline (and 95% confidence intervals) in serum total and LDL cholesterol in the tamoxifen (bold line) and control (dotted line) groups (ANOVA repeated measurements).

Notably, already after 3 months of tamoxifen therapy both total and LDL cholesterol levels had decreased even below the baseline levels measured before the chemotherapy: total cholesterol had decreased −4.6% and LDL cholesterol −8.3% below the baseline levels (*P*<0.0001 and 0.0001, respectively) (Figure 1

).

### Effect of clodronate on lipid levels

Totally, 19 patients were treated with intravenous clodronate in addition to adjuvant chemotherapy. Clodronate did not have significant effects on serum lipids (data not shown).

## DISCUSSION

In line with previous findings, adjuvant chemotherapy caused ovarian dysfunction (amenorrhoea or irregular menstruation) in the majority of patients in the present study. The risk of amenorrhoea was age-related, the older the women the higher was the risk of premature menopause after chemotherapy. As we have reported previously (Saarto *et al*, 1996a), the changes in serum total and LDL cholesterol correlated significantly with menstrual function after chemotherapy. The total and LDL cholesterol levels increased significantly only in patients who developed ovarian dysfunction while no changes were seen in patients who preserved regular menstruation. In the present study, HDL cholesterol remained unchanged in the patients with ovarian dysfunction while it even increased in our previous study.

In the present study, adjuvant tamoxifen therapy initiated after chemotherapy decreased the increased concentrations of total and LDL cholesterol. Already after 3 months of tamoxifen therapy, the total and the LDL cholesterol levels had decreased −4.6 and −8.3% below the baseline levels before chemotherapy, while total and LDL cholesterol remained at increased levels in the control group. The HDL cholesterol levels did not change in either tamoxifen or control group.

Tamoxifen treatment is associated with decreases in serum total and LDL cholesterol. This has been true especially in postmenopausal women (Love *et al*, 1990; Grey *et al*, 1995; Saarto *et al*, 1996b) but also to some extent in pre- and perimenopausal women (Caleffi *et al*, 1988; Powles *et al*, 1994). The effects of tamoxifen therapy on HDL cholesterol have varied across previous studies and have mostly been marginal. Retrospective studies have suggested a reduction of cardiac morbidity among tamoxifen users (McDonald and Stewart, 1991; Rutqvist and Mattsson, 1993), which has been related to the cholesterol-lowering effect of antioestrogens. However, a large double-blind, randomised, placebo-controlled NSABP Breast Cancer Prevention Trial (BCPT) reported that prophylactic tamoxifen did not influence cardiovascular risk in 13 388 women (Fisher *et al*, 1998). When the study population was further divided to those with high or low risk for cardiovascular events, tamoxifen was not associated with beneficial or adverse cardiovascular effects in either group (Reis *et al*, 2001).

Dyslipidaemia is an independent risk factor of CHD in both men and women. Low-serum HDL cholesterol levels seem to be even a stronger predictor of cardiovascular mortality than elevated LDL cholesterol levels in postmenopausal women (Bass *et al*, 1993). Cholesterol-lowering therapy with statins reduces cardiovascular events (Scandinavian Simvastatin Survival Study Group, 1994; Sacks *et al*, 1996; Long-term Intervention with Pravastatin in Ischaemic Disease (LIPID) Study Group, 1998) and mortality rates (Scandinavian Simvastatin Survival Study Group, 1994).

Hormone replacement therapy lowers total and LDL cholesterol and increases HDL cholesterol concentrations. Observational studies have found lower rates of CHD in postmenopausal women who use oestrogen as compared to nonusers (Stampfer and Colditz, 1991; Grady *et al*, 1992). However, two large intervention trials (HERS and WHI) found no benefit of oestrogen–progestin treatment on the risk of cardiovascular events (Hulley *et al*, 1998; Manson *et al*, 2003). Oestrogen plus progestin did not inhibit disease progression among women with established CHD and in fact an early increase in the risk of CHD events was noted in the HERS trial (Hulley *et al*, 1998). In the WHI trial, oestrogen plus progestin did not confer cardiac protection and actually increased the risk of CHD among generally healthy postmenopausal women, especially during the first year after the initiation of hormone use (Manson *et al*, 2003).

We conclude that adjuvant tamoxifen therapy reverses the adverse effects of chemotherapy on total and LDL cholesterol and lowers their serum levels even below the baseline. The serum HDL cholesterol levels, however, remained unchanged after chemotherapy followed by tamoxifen. The clinical implications of these findings still need to be studied as many factors other than serum cholesterol levels affect the risk of cardiovascular disease.

## References

[bib1] Bass KM, Newschaffer CJ, Klag MJ, Bush TL (1993) Plasma lipoprotein levels as predictors of cardiovascular death in women. Arch Intern Med 153: 2209–2216 8215724

[bib2] Bianco AR, Del Mastro L, Gallo C, Perrone F, Matano E, Pagliarulo C, De Placido S (1991) Prognostic role of amenorrhea induced by adjuvant chemotherapy in premenopausal patients with early breast cancer. Br J Cancer 63: 799–803 2039706 10.1038/bjc.1991.177 PMC1972375

[bib3] Bines J, Oleske DM, Cobleigh MA (1996) Ovarian function in premenopausal women treated with adjuvant chemotherapy for breast cancer. J Clin Oncol 14: 1718–1729 8622093 10.1200/JCO.1996.14.5.1718

[bib4] Bonadonna G, Valagussa P, Rossi A, Tancini G, Brambilla C, Zambetti M, Veronesi U (1985) Ten-year experience with CMF-based adjuvant chemotherapy in resectable breast cancer. Breast Cancer Res Treat 5: 95–115 3839424 10.1007/BF01805984

[bib5] Caleffi M, Fentiman IS, Clark GM, Wang DY, Needham J, Clark K, La Ville A, Lewis B (1988) Effect of tamoxifen on oestrogen binding, lipid and lipoprotein concentration and blood clotting parameters in premenopausal women with breast pain. J Endocrinol 119: 335–339 3199064 10.1677/joe.0.1190335

[bib6] Decensi A, Robertson C, Rotmensz N, Severi G, Maisonneuve P, Sacchini V, Boyle P, Costa A, Veronesi U (1998) Effect of tamoxifen and transdermal hormone replacement therapy on cardiovascular risk factors in a prevention trial. Italian Chemoprevention Group. Br J Cancer 78: 572–578 9744493 10.1038/bjc.1998.542 PMC2063049

[bib7] Del Mastro L, Venturini M, Sertoli MR, Rosso R (1997) Amenorrhea induced by adjuvant chemotherapy in early breast cancer patients: prognostic role and clinical implications. Breast Cancer Res Treat 43: 183–190 9131274 10.1023/a:1005792830054

[bib8] Diel IJ, Solomayer EF, Costa SD, Gollan C, Goerner R, Wallwiener D, Kaufmann M, Bastert G (1998) Reduction in new metastases in breast cancer with adjuvant clodronate treatment. N Engl J Med 339: 357–363 9691101 10.1056/NEJM199808063390601

[bib9] Early Breast Cancer Trialists' Collaborative Group (1992) Systemic treatment of early breast cancer by hormonal, cytotoxic, or immune therapy. 133 randomised trials involving 31 000 recurrences and 24 000 deaths among 75 000 women. Lancet 339: 1–15 71–85 1345869

[bib10] Early Breast Cancer Trialists' Collaborative Group (1998a) Polychemotherapy for early breast cancer: an overview of randomised trials. Lancet 352: 930–942 9752815

[bib11] Early Breast Cancer Trialists' Collaborative Group (1998b) Tamoxifen for early breast cancer: an overview of the randomised trials. Lancet 351: 1451–1467 9605801

[bib12] Fisher B, Constantino JP, Wickerham DL, Redmond CK, Kavanah M, Cronin WM, Vogel V, Robidoux A, Dimitrov N, Atkins J, Daly M, Wieand S, Tan-Chiu E, Ford L, Wolmark N (1998) Tamoxifen for prevention of breast cancer: report of the National Surgical Adjuvant Breast and Bowel project P-1 Study. J Natl Cancer Inst 90: 1371–1388 9747868 10.1093/jnci/90.18.1371

[bib13] Friedewald WT, Levy RI, Fredrickson DS (1972) Estimation of the concentration of low-density lipoprotein cholesterol in plasma, without use of the preparative ultracentrifuge. Clin Chem 18: 499–502 4337382

[bib14] Fukami K, Koike K, Hirota K, Yoshikawa H, Miyake A (1995) Perimenopausal changes in serum lipids and lipopoteins: a 7-year longitudinal study. Maturitas 22: 193–197 8746876 10.1016/0378-5122(95)00927-d

[bib15] Grady D, Rueben SB, Pettiti DB, Fox CS, Black D, Ettinger B, Ernster VL, Cummings SR (1992) Hormone therapy to prevent disease and prolong life in postmenopausal women. Ann Intern Med 117: 1016–1037 1443971 10.7326/0003-4819-117-12-1016

[bib16] Grey AB, Stapleton JP, Evans MC, Reid IR (1995) The effect of the anti-estrogen tamoxifen on cardiovascular risk factors in normal postmenopausal women. J Clin Endocrinol Metab 80: 3191–3195 7593425 10.1210/jcem.80.11.7593425

[bib17] Hulley S, Grady D, Bush T, Furberg C, Herrington D, Riggs B, Vittinghoff E (1998) Randomized trial of estrogen plus progestin for secondary prevention of coronary heart disease in postmenopausal women. Heart and Estrogen/progestin replacement Study (HERS) Research Group. JAMA 280: 605–613 9718051 10.1001/jama.280.7.605

[bib18] Koyama H, Wada T, Nishizawa Y, Iwanaga T, Aoki Y (1977) Cyclophosphamide-induced ovarian failure and its therapeutic significance in patients with breast cancer. Cancer 39: 1403–1409 851940 10.1002/1097-0142(197704)39:4<1403::aid-cncr2820390408>3.0.co;2-8

[bib19] Long-term Intervention with Pravastatin in Ischaemic Disease (LIPID) Study Group (1998) Prevention of cardiovascular events and death with pravastatin in patients with coronary heart disease and a broad range of initial cholesterol levels. N Engl J Med 339: 1349–1357 9841303 10.1056/NEJM199811053391902

[bib20] Love RR, Newcomb PA, Wiebe DA, Surawicz TS, Jordan VC, Carbone PP, DeMets DL (1990) Effects of tamoxifen therapy on lipid and lipoprotein levels in postmenopausal patients with node-negative breast cancer. J Natl Cancer Inst 82: 1327–1332 2199681 10.1093/jnci/82.16.1327

[bib21] Ludwig Breast Cancer Study Group (1985) A randomized trial of adjuvant combination chemotherapy with or without prednisone in premenopausal breast cancer patients with metastases in one to three axillary lymph nodes. Cancer Res 45: 4454–4459 2862995

[bib22] Manson JE, Hsia J, Johnson KC, Rossouw JE, Assaf AR, Lasser NL, Trevisan M, Black HR, Heckbert SR, Detrano R, Strickland OL, Wong ND, Crouse JR, Stein E, Cushman M, Women's Health Initiative Investigators (2003) Estrogen plus progestin and the risk of coronary heart disease. N Engl J Med 349: 523–534 12904517 10.1056/NEJMoa030808

[bib23] Matthews KA, Meilahn E, Kuller LH, Kelsey SF, Caggiula AW, Wing RR (1989) Menopause and risk factors for coronary heart disease. N Engl J Med 321: 641–646 2488072 10.1056/NEJM198909073211004

[bib24] McDonald CC, Stewart HJ (1991) Fatal myocardial infarction in the Scottish adjuvant tamoxifen trial. The Scottish Breast Cancer Committee. BMJ 303: 435–437 1912833 10.1136/bmj.303.6800.435 PMC1670561

[bib25] Nabulsi AA, Folsom AR, White A, Patsch W, Heiss G, Wu KK, Szklo M (1993) Association of hormone-replacement therapy with various cardiovascular risk factors in postmenopausal women. The Atherosclerosis Risk in Communities Study Investigators. N Engl J Med 328: 1069–1075 8384316 10.1056/NEJM199304153281501

[bib26] Newnhamn HH (1993) Oestrogens and atherosclerotic vascular disease-lipid factors. Bailliere Clin Endocrinol Metab 7: 61–93 10.1016/s0950-351x(05)80271-x 8435058

[bib27] Padmanabhan N, Wang DY, Moore JW, Rubens RD (1987) Ovarian function and adjuvant chemotherapy for early breast cancer. Eur J Cancer 23: 745–748 10.1016/0277-5379(87)90272-0 3653192

[bib28] Powles TJ, Jones AL, Ashley SE, O'Brien ME, Tidy VA, Treleavan J, Cosgrove D, Nash AG, Sacks N, Baum M, McKinna JA, Davey JB (1994) The Royal Marsden Hospital pilot tamoxifen chemoprevention trial. Breast Cancer Res Treat 31: 73–82 7981459 10.1007/BF00689678

[bib29] Powles TJ, Paterson S, Kanis JA, McCloskey E, Ashley S, Tidy A, Rosenqvist K, Smith I, Ottestad L, Legault S, Pajunen M, Nevantaus A, Mannisto E, Suovuori A, Atula S, Nevalainen J, Pylkkanen L (2002) Randomized, placebo-controlled trial of clodronate in patients with primary operable breast cancer. J Clin Oncol 20: 3219–3224 12149294 10.1200/JCO.2002.11.080

[bib30] Pripp U, Hall G, Csemiczky G, Eksborg S, Landgren BM, Schenck-Gustafsson K (1999) A randomized trial on effects of hormone replacement therapy on ambulatory blood pressure and lipoprotein levels in women with coronary artery disease. J Hypertens 17: 1379–1386 10526897 10.1097/00004872-199917100-00004

[bib31] Reis SE, Costantino JP, Wickerham DL, Tan-Chiu E, Wang J, Kavanah M (2001) Cardiovascular effects of tamoxifen in women with and without heart disease: Breast Cancer Prevention Trial. J Natl Cancer Inst 93: 16–21 11136837 10.1093/jnci/93.1.16

[bib32] Richards MA, O'Reilly SM, Howell A, George WD, Fentiman IS, Chaudary MA, Crowther D, Rubens RD (1990) Adjuvant cyclophosphamide, methotrexate and fluorouracil in patients with axillary node-positive breast cancer: an update of the Guy's/Manchester trial. J Clin Oncol 8: 2032–2039 2230895 10.1200/JCO.1990.8.12.2032

[bib33] Rijpkema AH, van der Sanden AA, Ruijs AH (1990) Effects of postmenopausal estrogen–progesterone therapy on serum lipids and lipoproteins: a review. Maturitas 12: 259–285 2145495 10.1016/0378-5122(90)90007-s

[bib34] Rutqvist LE, Mattsson A (1993) Cardiac and thromboembolic morbidity among postmenopausal women with early-stage breast cancer in a randomized trial of adjuvant tamoxifen. The Stockholm Breast Cancer Study. J Natl Cancer Inst 85: 1398–1406 8350363 10.1093/jnci/85.17.1398

[bib35] Rzymowska J (1999) Effect of cytotoxic chemotherapy on serum lipid levels in breast cancer patients. Pathobiology 67: 129–132 10394133 10.1159/000028062

[bib36] Saarto T, Blomqvist C, Ehnholm C, Taskinen MR, Elomaa I (1996a) Effects of chemotherapy-induced castration on serum lipids and apoproteins in premenopausal women with node-positive breast cancer. J Clin Endocrinol Metab 81: 4453–4457 8954058 10.1210/jcem.81.12.8954058

[bib37] Saarto T, Blomqvist C, Ehnholm C, Taskinen MR, Elomaa I (1996b) Antiatherogenic effects of adjuvant antiestrogens: a randomized trial comparing the effects of tamoxifen and toremifene on plasma lipid levels in postmenopausal women with node-positive breast cancer. J Clin Oncol 14: 429–433 8636753 10.1200/JCO.1996.14.2.429

[bib38] Saarto T, Blomqvist C, Virkkunen P, Elomaa I (2001) Adjuvant clodronate treatment does not reduce the frequency of skeletal metastases in node-positive breast cancer patients: 5-year results of a randomized controlled trial. J Clin Oncol 19: 10–17 11134190 10.1200/JCO.2001.19.1.10

[bib39] Sacks FM, Pfeffer MA, Moye LA, Rouleau JL, Rutherford JD, Cole TG, Brown L, Warnica JW, Arnold JM, Wun CC, Davis BR, Braunwald E (1996) The effect of pravastatin on coronary events after myocardial infarction in patients with average cholesterol levels. Cholesterol and Recurrent Events Trial investigators. N Engl J Med 335: 1001–1009 8801446 10.1056/NEJM199610033351401

[bib40] Scandinavian Simvastatin Survival Study Group (1994) Randomised trial of cholesterol lowering in 4444 patients with coronary heart disease: the Scandinavian Simvastatin Survival Study (4S). Lancet 344: 1383–1389 7968073

[bib41] Schaefer EJ, Famon-Fava S, Cohn SD, Schaefer MM, Ordovas JM, Castelli WP, Wilson PW (1994) Effects of age, gender, menopausal status on plasma low density lipoprotein cholesterol and apolipoprotein B levels in Framingham Offspring Study. J Lipid Res 35: 779–792 8071601

[bib42] Stampfer M, Colditz G (1991) Estrogen replacement therapy and coronary heart disease: a quantitative assessment of the epidemiologic evidence. Prev Med 20: 47–63 1826173 10.1016/0091-7435(91)90006-p

[bib43] Stevenson JC, Crook D, Godsland IF (1993) Influence of age and menopause on serum lipids and lipoproteins in healthy women. Atherosclerosis 98: 83–90 8457253 10.1016/0021-9150(93)90225-j

[bib44] Subramaniam S, Marar T, Devi CS (1991) Studies on the changes in plasma lipids and lipoproteins in CMF treated breast cancer patients. Biochem Int 24: 1015–1024 1781779

[bib45] Writing Group for the PEPI Trial (1995) Effects of estrogen or estrogen/progestin regimens on heart disease risk factors in postmenopausal women. The postmenopausal estrogen/progestin interventions (PEPI) trial. JAMA 273: 199–208 7807658

